# Designing Interactive Experiences for Children with Cochlear Implant

**DOI:** 10.3390/s18072154

**Published:** 2018-07-04

**Authors:** Sandra Cano, Leandro Flórez-Aristizábal, César A. Collazos, Habib M. Fardaoun, Daniyal M. Alghazzawi

**Affiliations:** 1 Multimedia Program, Universidad San Buenaventura Cali, 760032 Cali, Colombia; 2 Systems Program, Universidad del Cauca, 190001 Popayán, Colombia; learistizabal@admon.uniajc.edu.co (L.F.-A.); ccollazo@unicauca.edu.co (C.A.C.); 3 Systems Program, Institución Universitaria Antonio José Camacho, 760032 Cali, Colombia; 4 College of Information Technology, Ahlia University Bahrain, 999 Manama, Bahrain; hfardonn@ahlia.edu.bh; 5 Faculty of Computing and Information Technology, King Abdulaziz University, 21577 Jeddah, Saudi Arabia; dghazzawi@kau.edu.sa

**Keywords:** interactive experiences, children with cochlear implant, technology, interactive systems

## Abstract

Information and Communication Technologies (ICTs) have grown exponentially in the education context and the use of digital products by children is increasing. As a result, teachers are taking advantage of ICTs to include mobile devices such as Tablets or Smartphones inside the classroom as playful support material to motivate children during their learning. Designing an interactive experience for a child with a special need such as a hearing impairment is a great challenge. In this article, two interactive systems are depicted, using a non-traditional interaction, by the following stages: analysis, design and implementation, with the participation of children with cochlear implant in the Institute of Blind and Deaf Children of Valle del Cauca, Colombia and the ASPAS Institute, Mallorca, Spain, who evaluated both interactive systems, PHONOMAGIC and CASETO. Positive results were obtained, showing that the use of real objects can greatly influence the environment in which children interact with the game, allowing them to explore and manipulate the objects supporting their teaching-learning processes.

## 1. Introduction

Currently, ICT have grown exponentially in the education context. Interest thus has arisen in including them in the classroom as support material for the teacher. Accessing a digital technology involves more than simply internet, computer, mobile or video games [1]. A report published by the UN in 2013 indicates that “ICT must go beyond issues of equipment availability and connectivity; it is necessary to move toward the matter of their uses—and their impact on learning” [2]. Technologies are thus being transformed, looking for new ways in which the user can interact. In the classroom, the teacher is aiming for the student to have a playful experience, making use of their space and other elements that allow them to interact with real objects and that can go beyond entertainment, with a more educational content.

To design an Interactive experience, meanwhile, is to take into account a set of disciplines, such as design, interaction and the end user. This means that design should be centered on the user, according to their needs. Children are using digital products more and more. A report by UNICEF [3] shows the different ways that children use technology. As such, children on having contact with technological elements will be motivated and that means they can interact with the system and the content for a longer time, because they are enjoying the experience.

Before making use of technology, children played with physical objects to learn a variety of skills. An interface with real or tangible objects thus seeks to let the child interact with a real, rather than simply digital environment, where it only appears to be natural to them. Interaction design has shown interest in the design of experiences in the manipulation of a physical model. This can be a powerful learning tool to deepen a variety of concepts and these practices can be effective in age, gender and skill level.

Tangible technologies can be defined as “user interfaces, where there is a physical reinforcement of a virtual function, where it serves as a functional manipulator” [4]. The term tangible refers to physical elements of tangible interfaces. Their function is to physically represent digital information. Therefore, when designing this type of interactive technologies that includes physical and digital elements, areas such as electronics and computing are involved. The term Internet of Things, meanwhile, enables things such as real objects to be interconnected with technology so that they can become interactive and connected [5]. Tangible interfaces that involve IoT technologies therefore form what is an interactive experience in a real-and at the same time digital-context.

However, designing an interactive experience for a child without limitations is quite different compared to a child with limitations since, depending on their limitation, the design of these systems can change in aspects related with learning style, communication and interaction and so forth.

In addition, it should be noted that children can be very easily distracted and lose interest in a very short time, especially if they have special needs in their learning or limitations such as a hearing impairment.

Yussef Hassan [6] explains that the experience for this type of user (children) is not only to take into account that they can have an enjoyable experience or interaction but that children have different skills and preferences. Design of these products thus requires an adaptation and adoption necessary to ensure an interactive experience for the child with limitation. If these children have hearing disabilities, an inclusive design philosophy ought to be adopted [7,8]. Inclusive design refers to designing products for a wider range of users, which tries to satisfy their needs, making it an accessible and usable design [9].

Research has shown that children who use technologies and have an enjoyable interactive experience show better language skills, intelligence, structural knowledge and so forth. [10]. However, traditional interaction is carried out in an individual way, where the user has a goal and to achieve it focuses on the interaction of the device. Interaction design thus depends on the degree of attention it is desired to give to the environment in order to improve communication between the user and the system. It is worth noting that Norman [11,12] has defined 6 design principles that are important to bear in mind when designing the interaction. These are: visibility, feedback, affordance, mapping, limitations and consistency. However, research studies found are oriented to children without limitations. As such, the design principles may differ, more so if they are children that have no auditory aid. However, if they do have such and are in the process of language acquisition, communication should be visual. Cano et al. [13] proposed a set of principles to design serious games for children with hearing disabilities for teaching in literacy, in which they propose a model grouped into three aspects: education (literacy), game mechanism and user profile.

In this new era of technology characterized by different ways to interact using non-traditional interfaces, the way children learn within the classroom is changing. For instance, mobile learning [14] has become more motivating and engaging than staring at a PC while sitting still.

The article has the following structure. In Section 2, auditory disability, the difficulties faced by children and the method used to teach reading and writing is described. Section 3 contains a description related to the design of interactive experiences aimed at children and some related work using interactive technologies. Section 4 presents the design proposal of an interactive experience for children with cochlear implant from the Institute of Blind and Deaf Children of Valle del Cauca, Colombia. In Section 5 presents a discussion and analysis about the results obtained. Finally a set of conclusions and future work is presented.

## 2. Auditory Impairment

Auditory disability is defined as an obstacle that makes it impossible to process linguistic information through the ear, which is a condition that prevents people from receiving sounds and generates problems in communicating with society. The sounds that are received from the ear are included, such as: low, high, distant, close and complex that a human being can receive, such as speech sounds [15].

Therefore, a child with a hearing disability needs to learn to communicate through written language and receive information through reading. The teaching of literacy becomes a great challenge for them and for teachers who must adapt learning strategies to develop and improve motivation of the children. For this reason, they need an interactive experience that allows the teacher and the child to interact in an educational environment and in turn motivate learning.

Children with hearing disabilities present several barriers, such as difficulties associated with oral or written sounds. If you are a child without a hearing aid you must communicate through writing, your challenge is greater in attempting to understand the meaning of words. If you have a hearing aid you must learn to identify sounds, since they are more difficult when they are weak sounds and you are required to discriminate what you hear, or when there is a lot of environmental noise, it is difficult to understand the meaning of words.

A child with hearing impairment who has a cochlear implant must learn to know each of the sounds that make up a word, to learn vocabulary and thus verbalize. Children from the Institute of Blind and Deaf Children of Valle del Cauca, Colombia have incorporated a learning method for the teaching of reading and writing, called the invariant method [16].

## 3. Interaction with Children

Interaction is a communication mechanism carried out with an interface to establish a dialogue between the user and the system. The response of the system is channeled through an interface. Shneiderman meanwhile emphasizes the concept of direct manipulation to activate the use of user interfaces [17]. It is therefore based on cognitive theory, human perception, rational decisions and human behavior.

Nowadays, mobile technologies are becoming very important, since they have two aspects that attract the attention of users: mobility and tactile interaction [18]. In children, tactile interaction is a motivating factor. In the educational area for the teacher, the portability characteristic has been of great interest for use within the classrooms. However, many existing mobile applications are not intended for children with special needs and more than integrate the method of teaching invariant method.

From an educational approach, reference is made to the theory of Piaget [19] called constructivism, in which he states that children learn best when they engage in construction of a meaningful product. Another study, by Vygotsky [20], is based on sociocultural learning, where children manage to acquire roles. It also refers to the symbolic game in which the child transforms an object and in his imagination turns it into another object that has a different meaning for the child. Moreover, Piaget thought that learning occurs through adaptation, where children adapt to the environment, where they build knowledge structures by means of experiences of the world and interaction with it.

In this new era, children are more exposed to technologies and advances in technology. Therefore, the use of interactive technologies in children is very common. Interactivity has been defined by Shneiderman [17], defining interactivity as a cyclical process in which two actors alternately think, speak and listen. The quality of the interaction depends on the quality of each sub-task.

Wilson & Sarin [21] meanwhile carried out a study that proposes a mobile device as physical medium to connect with other devices or hardware elements to exchange information. Therefore, this could be considered as an interaction technique that helps to improve interaction between user and system. In addition, Wellner et al. [22] cite the importance of increasing and enriching the real world with the digital one, providing functionality without forcing the user within the digital world.

## 4. Interactive Technologies

Digital spaces have been manipulated by traditional interaction mechanisms such as keyboard, mouse and joystick, which are used to control and manipulate representations displayed on output devices, such as a screen. With the increase in the use of technologies, these traditional ways of interacting are changing, seeking to integrate other non-traditional objects that act as input and allow interacting with the user. New forms of interaction that combine a physical and digital environment are therefore proposed.

Interaction with real objects using technology implies that the elements must be interconnected to communicate with the technological device (Tablet, Smartphone or computer). To connect everyday physical objects with technology requires knowing a set of electronic devices that can be interconnected through a variety of communication solutions such as Bluetooth, Wi-Fi and ZigBee. Therefore, IoT is necessary to capture information that is interconnected between Wi-Fi or Bluetooth and other devices. As indicated by Ng [23], each activity involves interconnection patterns of interaction with different things.

The type of interaction being proposed can thus be said to be a type of tangible interaction applied to IoT. This type of interaction paradigm could exploit different levels of user attention, which can change with the context of interaction. IoT objects can detect the external environment and collect sensitive information from the user and share it through the internet.

Electronic devices such as sensors or actuators are required to detect or capture the external environment. A sensor can refer to a device capable of detecting physical or chemical quantities and be transformed into electrical variables. Some of the variables that can therefore be found and measured are distance, pressure, resistance, movement and Radio Frequency Identification (RFID). An actuator is a device that is able to transform a specific type of energy into an activity in the process, in order to generate some effect on the external element. Furthermore, other electronic devices (loudspeakers, light emitting diodes, motors, etc.) can also be connected to the actuators to translate the information or as a feedback message, whether of a visual, auditory or haptic type, according to how the action has been received.

**Figure 1:**
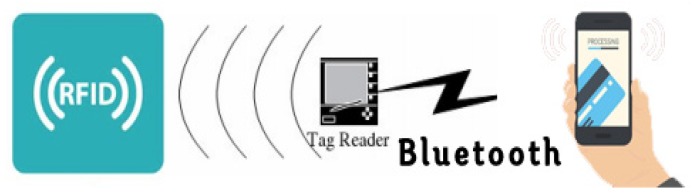
RFID System with Tags.

RFID sensors use radio waves to identify people or objects (Figure 1). The most common way they are used is that there is a unique number for each object labeled with an RFID tag [24]. Different authors have proposed a variety of potential applications using RFID technology in the design of interactive systems for children [24,25]. The work presented by Motoyoshi et al. [26] integrates RFID technology in an educational context for building blocks. A work presented by Valero & Adam [27] describes the advantages and limitations of RFID technologies, such as the limitations of interference with materials can affect their communication. Another work that has used RFID is presented by the University of Utah [28], an application of RFID for a care robot for visually impaired people. The system consists of a mobile robot and passive RFID tags placed in indoor environments.

Depending on their source of energy, RFID tags can be active, passive and semi-passive [25]. The Active tags have their own energy source and can actively transmit to the reader. Passive tags do not have a source of energy and use the request by radio of the reader to obtain sufficient energy to respond. The semi-passive tag has a power source to run the tag chip but uses the power source of the reader to communicate with the reader. One of the best-known, low cost RFIDs is the RFID-RC522. It uses a voltage of 3.3 volts and is controlled by means of the SPI (Serial Peripherical Interface) protocol. It is one of the most popular protocols for working with serial communication, due to its transmission speed, simplicity and operation. It is therefore possible to work with Arduino using the SPI protocol. RC522 uses a modulation and demodulation system at a frequency of 13.56 MHz. The card that comes with the RFID module has 64 memory blocks, giving these RFID tags a serial number that has five hexadecimal values.

**Figure 2:**
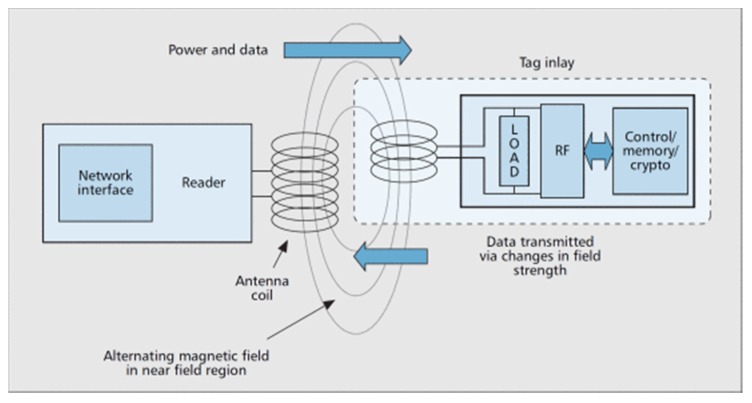
RFID Reader and Tag [29].

Communication between a reader and a tag is through a mechanism called load modulation. The RFID reader consists of a radio frequency module, a control unit and an antenna coil which generates high frequency electromagnetic field. While, the Tag is a passive component that consist of just an antenna and an electronic microchip, when it gets near to electromagnetic field of the transceiver, due to induction, a voltage is generated in it is antenna coil and this voltage serves as power for the microchip (Figure 2)

RFID technology combined with other devices, such as Arduino, facilitates the use of electronics and programming. Therefore, for this proposal for prototype number one, a nano-Arduino is used with an RFID reader to read a set of tagged objects. However, so that the data that is captured from the RFID reader can be sent from Arduino to a mobile application it is necessary to use a Bluetooth sensor for communication. RFID technology combined with other devices, such as Arduino, facilitates the use of electronics and programming. Therefore, for this proposal for prototype number one, is used a Nano-Arduino with an RFID reader is used to read a set of tagged objects. However, so that the data that is captured from the RFID reader can be sent from Arduino to a mobile application it is necessary to use a Bluetooth sensor for communication.

Another well-known sensor is a touch sensor using a push button. It works by contact with the user, so it has two states, pressed (1) and not pressed (0). So, on not being pressed it does not have any electrical contact, until it is pressed. This type of sensor can be supported by an actuator such as a light-emitting diode, which is integrated to give a visual response that the action was received. This type of response works as communication with the user but may change depending on the child’s limitation. That is, a child with a cochlear implant is in the process of acquiring language, so the response the child should receive must be visual using RGB diodes that emit light in a wide range of colors.

IoT technologies combine sensors and actuators to exchange and consume data with minimal human intervention. The connection of the IoT with these devices was revealed in a guide RFC-7452 [30] which describes four common communication models: Device-to-Device, Device-to-Cloud, Device-to-Gateway and Back-End Data-Sharing. These models highlight the flexibility with which IoT devices can connect. Therefore, the design of these systems is characterized so that the child can have a type of interaction that goes beyond the conventional.

## 5. Methodology

We took account of the philosophy of user centered design with children with cochlear implants. Therefore, the following stages are followed: analysis, design and implementation. Two proposals for designing Interactive Experiences using a non-traditional interaction are presented below.

### 5.1. Participants

The participants were children in the pre-kindergarten, transition and first levels at the Institute of Blind and Deaf Children of Valle del Cauca, that is, children between 7 to 11 years of age. Some of these children have a hearing aid, others have cochlear (INCSVC) implants, others are listeners and one girl has an additional motor and cognitive disability. Children from the ASPAS Institute (Mallorca-Spain) also participated in this case study. A consent form was signed by parents to be able to evaluate the children.

### 5.2. Analysis

To analyze the profile of the child with a cochlear implant, a questionnaire was administered to a number of teachers who were teaching reading and writing with the invariant method. It was thus observed that teachers work with physical resources to teach the invariant method. In addition, reference is made to the learning defined by Vygotsky, which states that learning is a process between the child and the environment is based on aspects of motivation corresponding to their cognitive development. The INCSVC children, when they are just learning to listen, tend to be more visual.

**Figure 3:**
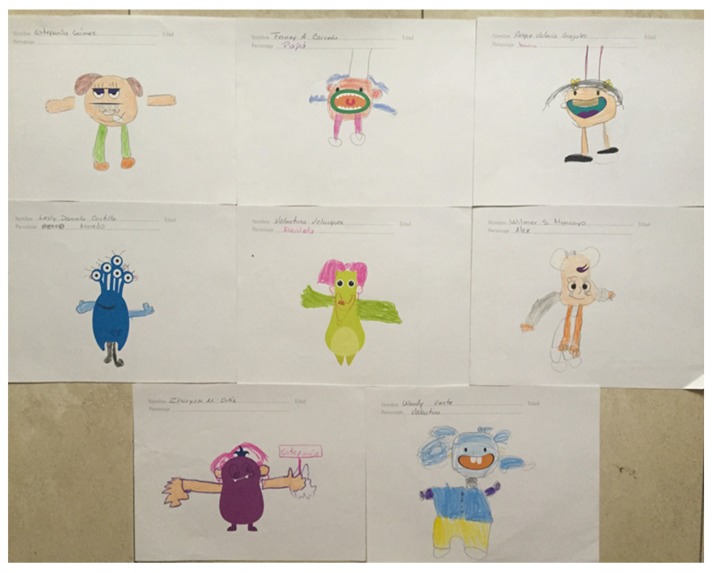
Drawing made by the pre-kindergarten children in the Institute of Blind and Deaf Children of Valle del Cauca.

Another determining factor of the child is the result of an activity that was carried out with them, in which they drew a certain character. The activity is described in a study by Cano et al. [31]. The results obtained in the drawings showed that the children knew each part of the body of a person, since this was the first thing that they drew (Figure 3); the use of the colors and the combination of these likewise. It is important to mention that one of the parts that no child was drawing were the ears or their aid device (hearing aid or implant). Moreover, these children were newly implanted and their language acquisition was very low, so they experienced difficulty when assigning a name to the character. Their name for the character was almost always related to words they already knew, for example: dad, mom, or proper names.

In order to identify aspects of children with cochlear implants, several different inquiry activities were carried out, such as interviews with the teachers and speech therapists, in order to capture information about the child. Observations of the therapies with the children and the various activities involving the children were also made. These may vary depending on the support they require [32,33].

Through the drawing we were able to identify their favorite colors. It was difficult for them to assign a name to the character, because they were in the process of acquiring a language. So, they put the first thing that occurred to them: the name of their father or mother, classmate, the psychologist, among others. Correct use of colors and the combination of them was also observed. However, it was difficult to draw the shape of the hands or feet. They were drawn very small or very large but not proportional to the body.

### 5.3. Design

Based on the analysis collected about children with a cochlear implant, it was decided to motivate the children through handling real objects that integrate IoT technology. The defined aim for this educational experience is that the child can distinguish between vowel and consonant sounds and learn the correspondence of each sound with the linguistic sign.

The proposed design, it is worth noting, is based on user-centered design, which emphasizes the contribution that understanding user diversity makes to informing these decisions. In 2005, the British Standards Institute defined that taking inclusive design into account is “the design of mainstream products and/or services that are accessible to and usable by, as many people as reasonably possible, without the need for special adaptation or specialized design”.

Described below are two proposals that came to fruition based on analyzing the profile of the child, in the context of education, the first prototype as an aid in literacy learning and the second for learning music.

#### 5.3.1. PHONOMAGIC

**Figure 4:**
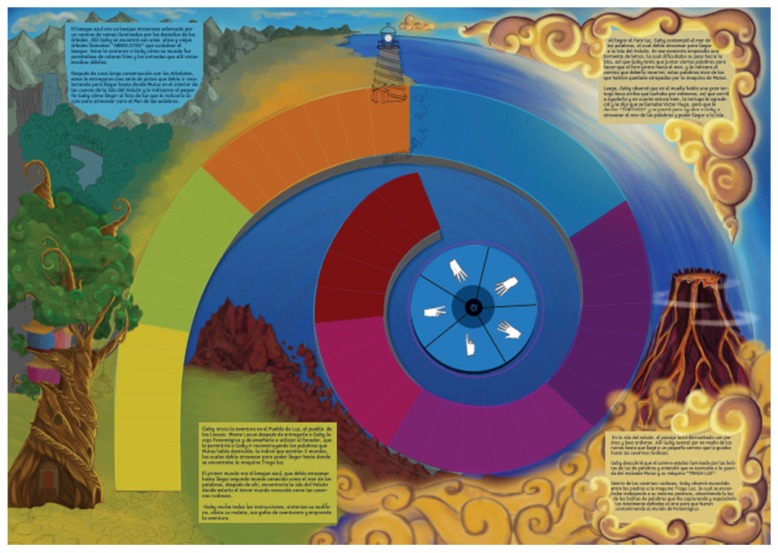
First prototype of the PHONOMAGIC physical board.

The first proposal is PHONOMAGIC (in Spanish, Fonomágica), a physical board that integrates non-traditional interaction using IoT technologies by means of sensors. For the design of the board, the anatomy of the inner ear is proposed, where aspects of the game are related and each level is associated with the levels of the ear (external, middle and internal). Each level has two associated sub-levels and a story and may be viewed as a cochlear labyrinth (Figure 4).

The story of Phonomagic has two main characters, Gabriel and Gabriela. There is also an enemy character called Mutus, who steals words from the children. Moma Locua, who helps Gabriel or Gabriela on the Phonomagic journey, delivering a range of powers with which to deal with obstacles. Moreover, the “Magic Box” is a magical element that helps to recover light spheres, thus helping Gaby recover words.

**Figure 5:**
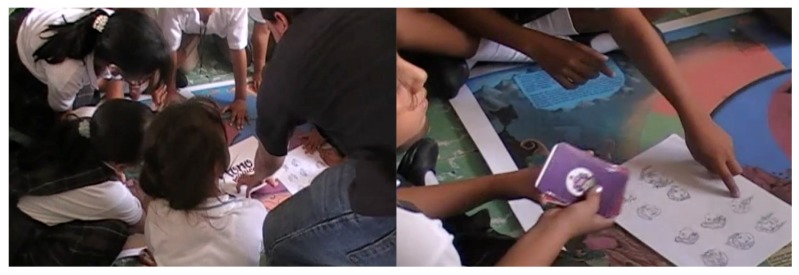
Children participating in the design.

It is also important to mention that this design was participatory (Figure 5), in which the opinions of the children were taken into account in such aspects as the Phonomagic logo, character design, colors of the board and the design of the cards.

**Figure 6:**
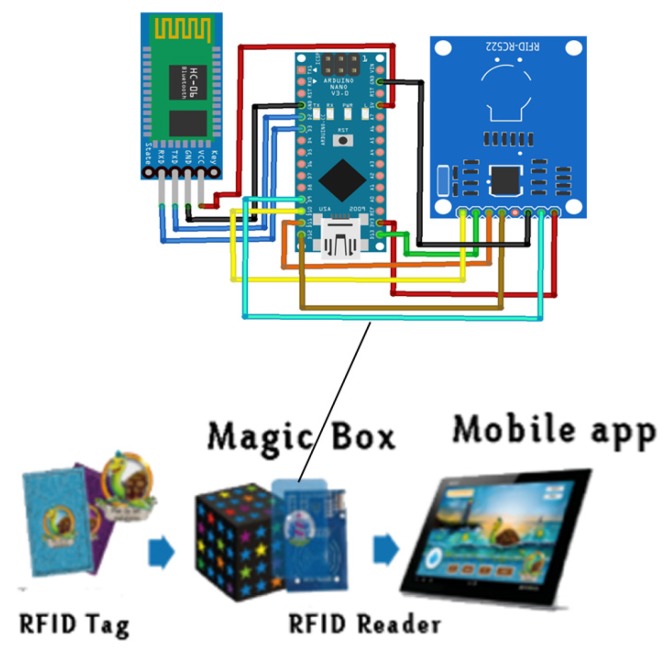
Interaction phono-cards with RFID + Tablet.

Each level and sub-level is represented by a color and have a card as a means of interaction with the Tablet (Figure 6). To conduct such a type of interaction with real objects (color cards), an RFID tag is added to each card. Therefore, an RFID reader is needed that can identify each of these unique codes assigned to the cards. An RFID-RC522 sensor is used, which is responsible for reading by Radio Frequency each of the labels incorporated in the internal part of each card. This is connected to a nano-Arduino using the SPI protocol, which operates at a frequency of 13.56 MHz, having a reading range that can be from 10 m to 100 m. The RFID cards used are passive cards, so their reading range is reduced to centimeters. Also, the RFID tags used are read-only, that is, the unique code (IDENTIFIER) they contain cannot be modified.

The connectivity used for this type of interaction with IoT technologies is Device-to-Device. Connection is made between the nano-Arduino and the mobile device (Tablet) via Bluetooth. As such, the electronic components are implemented inside a box that has the shape of a “magic box” according to the PHONOMAGIC story. The “magic box” inside the story, is a magical element that is inherited to the main character of the game, called Gaby, which allows to retrieve words that had been destroyed by the machine of light wallow.

The mobile application has a SQLite database. This stores the identifier information of each RFID tag, which corresponds to each color of the card that has a level associated with it. This is carried out also to monitor each activity.

#### 5.3.2. CASETO

The second prototype is called CASETO [34]. Its name comes from the fact that the design was made in the form of a cassette but it has the functionality of supporting children with cochlear implants in music teaching. A physical system that interacts by means of a Tablet was therefore designed. The physical system communicates with the Tablet using a type of Device-to-Device connectivity, by means of an ARDUINO-UNO and mobile device (Tablet), using Bluetooth.

**Figure 7:**
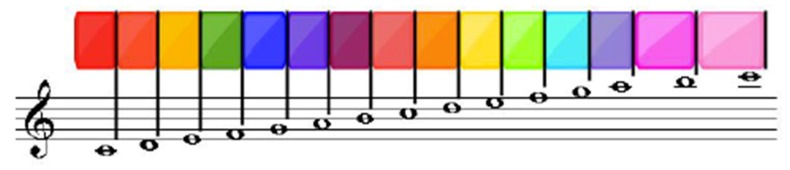
Colors associated with musical notes.

An interactive piano is proposed as a design, in which each musical note is visually represented by a color. Color tests were carried out to determine the scale of colors to be handled according to the musical notes. According to the observations obtained, it was decided to implement the colors according to the chromatic scale, starting with red to represent Doh, orange Re, yellow Mi, green Fah, blue Soh, purple Lah and violet Ti. When the child presses on a note, the color associated with that note will be projected on the respective key (Figure 7).

**Figure 8:**
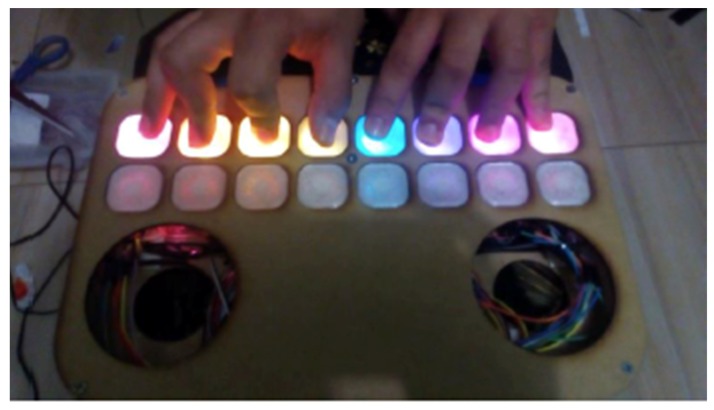
CASETO System.

The action of pressing each note corresponds to a tactile sensor, which corresponds to an electronic element called a button. It has two states, pressed (1) and not pressed (0). When a note is pressed, the response of the action is that the keyboard emits light of the color corresponding to the musical note. For this, a light-emitting diode, called an RGB diode, was used as an actuator. The connectivity used is Device-to-Device, between the arduino and the mobile application using Bluetooth communication. Moreover, a pair of speakers that will serve to amplify the audio of each musical note is used as an actuator (Figure 8).

### 5.4. Implementation

Because this implementation was to work with low-cost technologies, it was decided to make the mobile application for Android operating systems, which contains each of the activities corresponding to a physical system applying IoT technologies.

Implementation of the two proposed prototypes is described below.

#### 5.4.1. PHONOMAGIC

##### Playing the Game

**Figure 9:**
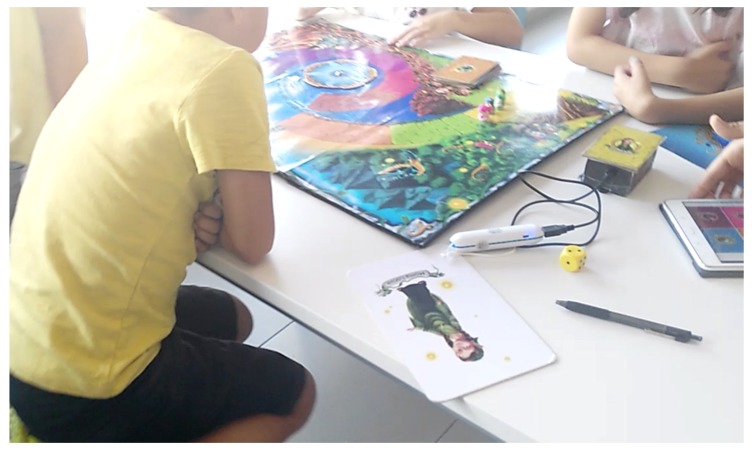
Playing PHONOMAGIC at the ASPAS Institute in Mallorca, Spain.

The physical game is designed to be played by 2 to 6 players. Players gather around the game board to start the game. Each child is assigned a token of a respective color. The chips represent the main character, Gaby. Each child takes their turn with the spinner located on the board to obtain a number of steps that must be advanced along the squares on the board. On reaching a certain box, the player will be assigned a card with the respective color of the box, which must have contact with the magic box to be able to read the code of the RFID tag. The code is then sent via Bluetooth to the Tablet, where depending on the card, the child must perform an activity (Figure 9).

Two evaluations of Fonomagica were carried out, in which a set of variables that would help to measure the functionality of the game were defined. We thus selected the following variables to measure: number of errors, number of failures and the time taken to perform the activity. The first evaluation was carried out in the Institute of Blind and Deaf Children of Valle del Cauca and the second evaluation with the ASPAS Institute in Mallorca, in Spain.

**Figure 10:**
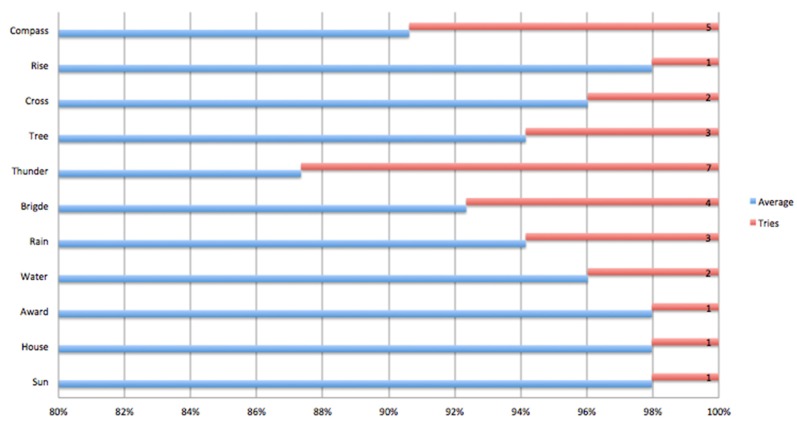
Evaluating PHONOMAGIC in the INCSVC.

The first evaluation measured variables that might validate whether or not the words associated with each level were the correct ones (Figure 10). This evaluation was carried out with the children of the Institute of Blind and Deaf Children of Valle del Cauca. The first level, which corresponds to the yellow card, contains words such as *cross*, *tree* and *climb*, while the green card contains the words *water*, *rain*, *bridge* and *thunder* (vertical axis). The children found some difficulty with words comprising two consecutive consonants or two consecutive vowels, or both, such as *thunder* and *cross*. In Figure 10, the word thunder can be seen to have the highest number of attempts (horizontal axis), followed by *compass* and *bridge*. It is also worth pointing out that these are not very common words in everyday use. The word *sun* presented least trouble. It is a monosyllabic word and has a syllabic combination of consonant-vowel. It should also be borne in mind that the words were selected according to the scenario of the game. However, for transitional children and first-graders these types of words are difficult.

From the results obtained along with the teacher, it was decided to adjust each level according to the number of syllables of a word.

**Figure 11:**
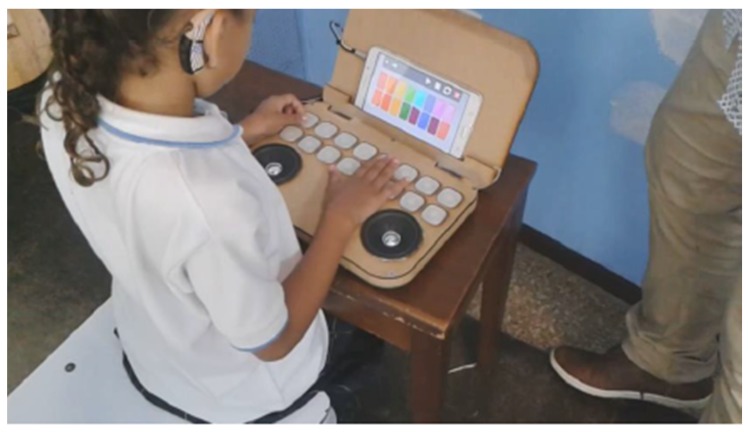
Design of CASETO for the Institute of Blind and Deaf Children of Valle del Cauca, Colombia.

In the second evaluation (Figure 11) carried out with children in the ASPAS Institute in Mallorca, they were asked some related questions; such as (1) Did you understand the activity? (2) Did it need a lot of effort? (3) Did you have fun? (4) Did you have to concentrate hard? Only six evaluations were undertaken, as the six children played at the same time. Most enjoyed the experience and only one nine-year-old girl had trouble because in addition to having a cochlear implant she had a motor problem and mild language retardation. However, even though the application featured audio support in the pronunciation of the word, very often the children could not hear clearly and there was always a need to support them. It was also observed that there were children competing for who would be the first to reach the end of the road. However, regardless of whether or not the phoneme sequence was constructed correctly to form a word, the children all encouraged their classmate to achieve it. The game also helped with counting, whenever a player did not know the number that followed, since before going on to do the activity, each player must carry out the counting of blocks that the character is required to advance.

PHONOMAGIC, an interactive system beyond entertainment but with a playful content associated with the educational context, can serve as a support tool in the classroom for the teacher and when interacting in a real environment that interconnects with a digital item, motivating children more to want to be linked to that experience.

RFID tags are similar to a sticker that can be attached to, or incorporated into, a product. With the PHONOCARDS, the RFID tag was incorporated into each card. It was observed, however, that it was sometimes difficult to read the tags. As the response was not immediate, therefore, the children became frustrated. The problem is related to the fact that the RFID reader was hidden inside a black box and the type of material sometimes hindered the sending of effective communication waves by the RFID Card. What is being tagged is also important therefore, as well as where to place the tag so that it can be easy to read and within range of the RFID reader.

#### 5.4.2. CASETO

CASETO is a non-traditional type of interaction (Figure 11) that uses a number of electronic components such as push-buttons, activated when pressed with a finger. The RBG LED diodes and speakers are used as a feedback of the action performed by pressing a button. With the LED diodes, feedback is visual, while with the two speakers it is auditory. An ArduinoMega was used to capture each one of the states of the 16 buttons that represent a musical note. So the information is captured through the digital ports of the Arduino and sent by Bluetooth to the mobile application. The type of connectivity that was used was Device-to-Device through a Bluetooth communication. The type of sensor that was used was a tactile sensor and, as feedback, light emitting diodes were used, which is integrated to give a visual response of the received action and a pair of speakers to amplify the audio. This type of response works as communication with the user but may change depending on the child’s limitation.

The mobile application has two modes of interaction with the child, guided mode and free mode. Free mode allows the child to press the buttons as he wishes and experiment with the piano. While the guided mode includes two songs—*Happy Birthday* and *Sunflowers*. On selecting either of them, it begins to show the sequence to be followed in the application. In this guided mode, visual-motor coordination is worked on, since the child needs hand, eye and finger coordination to press the correct button. However, since the application has all the buttons in the corresponding color and in the physical system all of them are white and their color is revealed only when pressed, the children had difficulty making the association of what they see in the application with the physical piano. In addition, many children, seeing that the speakers were uncovered, were afraid that it might cause them harm. However, when interacting with the piano they were motivated, since they had a visual stimulation.

In the design, each button on the piano was made with a 3D printing material but the material was very resistant, so to press each button the child had to use quite a lot of force. In addition, since all the buttons were printed in white it was more difficult for them to press the correct key when the mobile application showed them the guided sequence to follow. One positive aspect was that in the communication of each of the tactile sensors, the feedback response was immediate.

## 6. Discussions

Both proposals presented are related to the design of tangible interfaces using IoT technologies, demonstrating that everyday objects are more and more being connected with technologies. In turn, tangible interfaces are being used more and more, so that it could be said that IoT is transforming the interactions. With every advance we are getting closer to the vision of Weiser of located computing [35], where computers connect through everyday environments. Tangible interaction furthermore is a natural facilitator for learning and collaboration [36]. These physical artifacts are new input devices, which can be attached to virtual objects for manipulation or for expressing action. Hourcade [37] conducted a study related to interaction design for children, taking into account design principles related to visual design, interaction styles and the use of input devices and these can be adjusted according their needs. Hourcade also states that in the field of interaction design oriented to children, researchers are taking into account theories such as constructionism by Piaget and Vygotsky with socio cultural approaches and others concerned with information-processing.

For the design of these proposed systems, PHONOMAGIC and CASETO, a process has been followed that initially involves identifying a set of aspects, such as the profile of the child with a cochlear implant, the context of use and the pedagogical objectives to be achieved. To analyze the profile of the child, a set of activities were carried out, to capture information about the child, such as drawings, interaction with the Tablet, building a character from body parts and building a story with characters. All this was done with the purpose of capturing the interests and the opinion of the child about the characters and story to be part of PHONOMAGIC. However, the children who were evaluated had limitations in language due to lack of vocabulary, so they could not capture verbal information since their oralization is very low; they do not understand many words when they are asked questions. The activities that were carried out therefore had a lot of visual accompaniment and were related to activities conducted in class. For example, through drawing and the colors used, the colors that might be involved in the design of the PHONOMAGIC board were determined, such as warm colors and preferably in living tones; the type of characters that the story should include were also determined. Due to the children being very young and having an auditory limitation, they did not show much imagination with the characters. When a non-human character was presented to them, most of them could not distinguish what it was or did not identify with it. From this analysis, it was decided to choose a character with a cochlear implant. Then, making a connection to the genre, they were presented with the male character called Gaby but the girls asked Why not a girl? It was therefore decided to create two characters using both genders, male and female, so that when it is a boy, he is called Gabriel and when it is a girl, she is called Gabriela. An enemy was also created for our main characters, whose name is Mutus. His purpose is to steal sounds from words. Furthermore, some words were changed in the PHONOMAGIC story because there is a lot of vocabulary that the child does not know but that can be read as a story in the company of the teacher to have a support for language and comprehension. A user interaction was thus designed whose purpose was individual but at the same time that the child could interact with in a collaborative manner. That is why the board involves 2 to 6 players.

In designing the interaction, the first proposal was that the card could be read with a QR code. However, the interaction was not very real, nor did it have a relationship with the story. When we evaluated the different words of the story and the comprehension, the children worried about what a phonogram was, what the box contained and why it was magical. From these concerns and curiosities presented by the children, it was decided that the interaction must be through a magic box that would be responsible for producing words through the Tablet and whose interaction would be tactile, and the child can correctly construct the word to be assigned, so Gaby can acquire each of those sounds stolen by his enemy, Mutus. The decision was also taken to involve a physical and digital interaction based on the desire to allow children to develop collaborative skills among classmates, without relying on the teacher but on themselves. The articulation of a team thus allows children to acquire knowledge altogether, in such a way that they can guide and support each other to carry out the game. It is important to mention that from the construction of the story and interaction with PHONOMAGICA, work is being done on constructing interactive stories for deaf children [38].

For the second proposal, the CASETO tool was developed to teach music to children with hearing disabilities. Interaction with music is not only made in an auditory way but can be expressed visually or using other senses such as touch through vibrations. So for this proposal the research was based on Christine Sun Kim, a deaf woman from birth who relates sound to art [39]. With CASETO we wanted to start with something basic that supports children in the teaching of music and musical notes. The children of the INCSVC take a Music course in which they are taught the musical notes, the intonation or a rhythmic pattern to be followed by using colors. A set of activities were also performed, such as associating a musical note with a color, or while listening to a symphonic melody they would draw. It was observed that children can differentiate shades of colors, that is, they can tell the difference between a light blue and a dark blue. So, the decision was made to represent each musical note associated with a color, relating the colors and tones with the musical note, that is, a C Major is represented by a dark red color and a C minor by a light red color. The interaction was intended to be totally physical, in such a way that they could experience the interaction as if it were a real musical instrument, where a musical note accompanied by a color represents each button of the instrument. The mobile application acts as a notebook of activities that guides the child in their interaction. That is why it has two modes of interaction with the application, which relate to two levels of difficulty. The free mode allows the child to experiment with the piano, to understand the interaction and at the same time to create his own music, while the guided mode consists of following the sequence of musical notes of a certain song. However, when the guided mode was evaluated, the children had difficulty following the sequence, since they said they could not relate what was in the physical instrument (buttons that had no color while they were not pressed), with the mobile application that had colored buttons to be pressed (Figure 8). This was very difficult for them, so when they were asked if they had to focus too much on the activity, they responded affirmatively. The evaluation that was carried out with the INCSVC children was subjective, due to their lack of vocabulary for understanding sentences. It was therefore a direct observation evaluation when interacting with CASETO, where eight children (four boys and four girls) participated. Seven out of the eight children had a cochlear implant and one girl had no hearing aid, so her communication system was lip-facial reading. Both groups of children, from ASPAS and INCSV, had difficulties with the buttons, since the material of the keys was very rigid, so they had to press hard. Once they received the visual feedback that the button was lighting with an associated color, it motivated them a lot to continue experimenting with the instrument. The speakers of the tool were exposed, which caused some children to be afraid, thinking that it would harm them but for the girl who did not have a cochlear implant, it was a positive thing because she could feel the vibrations of each musical note, and explored the vibrations more compared to the other children who did have a cochlear implant. A free interaction test was conducted to measure the average time children could spend browsing the interactive system. The average was 2 min and 13 s but mainly because the rest of the children were waiting to interact with the system again. They are also children who are very dependent on the teacher. Usually when they were told to experiment the system freely, most of them felt self-conscious and evaluated, it was like they were expecting instructions. In the guiding mode, some children could not follow the sequence and felt frustrated and did not want to continue doing the activity. It was observed that the manipulation part of the instrument could help the child in visual and motor coordination. Therefore, the interactive experience [40,41] cannot involve only an educational context but a context that is also therapeutic like CASETO or PHONOMAGIC with an educational content in literacy teaching but at the same time it had a mathematical content because they had to count on advancing to each box and a social content because they had to interact with the other classmates and could support each other.

## 7. Conclusions and Future Work

RFID systems are on the way to including a change in the new paradigm of communication and information technologies, in such a way that not only people and devices will be interconnected to worldwide networks but other traditional objects. In addition, it is observed that a simple implementation that does not require much cost can help to generate an interactive experience in children, with the purpose of motivating them during their learning. The type of interaction that has been implemented shows that by integrating a real environment with digital elements, children have fun and are involved with others which is positive for them, because they feel they are playing and not that they are being evaluated.

One positive aspect is that, initially, there was a rule of the game that the child who did not perform the activity of following the path correctly on the Tablet had to give up the turn and allow the next classmate to play. However, although the children were competing, they did not take it that way, and they all supported the classmate who was struggling, since the teacher was not part of that teaching process but the children themselves.

Therefore, the use of real objects can greatly influence the environment in which the child develops in the game. Tangible interfaces, as they are known, promote interactivity using real objects that allow the child to explore and manipulate the objects for their learning.

In the design of interactive experiences using tangible interfaces applied with IoT technologies, it is important to find the right balance between interaction styles that are found, taking into account the type of application and the context of the interaction, since these interactions may change depending on the needs of children with limitations and if they are unconventional and at the same time complex, they can produce a more learning curve.

It is also important to mention just how the different tangible aspects, these being the physical, multi-sensorial and dynamic elements, are better able to support the child on obtaining better feedback using the senses (visual, auditory and tactile), enabling the child to carry out an exploration of their own learning.

As future work, we want to improve the mobile application and make some adjustments to the CASETO physical piano, such as covering the speakers and changing each button to the respective color of the musical note. In PHONOMAGIC, the levels for the transition grades are being adjusted, first for the Institute of Blind and Deaf Children of Valle del Cauca.

It is also important to establish a guide for the design of interactive experiences aimed at children with special needs using tangible interfaces applied to the IoT. In this guide, it is important to describe key parameters such as connectivity, data exchange, suitable sensors and a set of actuators that can be applied to the design of these interactive systems for children with hearing problems.
